# HaNDL Syndrome Presenting with Thunderclap Headache

**DOI:** 10.1155/2021/9925004

**Published:** 2021-05-31

**Authors:** Melvin Parasram, Ashwin Malhotra, Andrea S. Yoo, Saad A. Mir

**Affiliations:** NewYork-Presbyterian Hospital/Weill Cornell Medical Center, Department of Neurology, New York, New York, USA

## Abstract

**Introduction:**

Transient headache and neurologic deficits with cerebrospinal fluid lymphocytosis (HaNDL) is defined as a secondary, nonvascular headache disorder characterized by the findings described in its name. Patients with HaNDL syndrome typically present with gradual onset migrainous headaches of moderate to severe intensity with transient neurological symptoms. *Case Report*. We discuss a patient who presented with thunderclap headache, recent transient neurologic deficits, and was ultimately diagnosed with HaNDL after an extensive neurologic evaluation.

**Conclusion:**

Thunderclap headache has very rarely been described in patients with HaNDL. After excluding emergent and secondary causes, HaNDL should be considered in patients with thunderclap-quality headaches, particularly when there is a history of transient neurological symptoms.

## 1. Background

Transient headache and neurologic deficits with cerebrospinal fluid lymphocytosis (HaNDL) is a rare, benign, and self-limiting syndrome characterized by moderate to severe headaches, stroke-like deficits, and cerebrospinal fluid (CSF) lymphocytosis [1, 2]. Patients with HaNDL develop neurologic deficits such as parasthesias, aphasia, altered mental status, and weakness. Migrainous type headaches of moderate to severe intensity often lag behind these focal symptoms [3–5]. Duration of neurological deficits can range from minutes to hours, and repeat attacks can occur for days to weeks following the initial event [2, 4]. The underlying pathophysiology is not fully elucidated, although a viral or inflammatory etiology has been suggested given the CSF lymphocytic pleocytosis [1, 2].

HaNDL often present to the emergency room with neurological emergencies since the stroke-like symptoms are alarming and severe. Computed tomography (CT) and magnetic resonance imaging (MRI) are unremarkable while perfusion studies can demonstrate focal hypoperfusion in symptomatic areas [4, 6]. Electroencephalography (EEG) is typically negative or can occasionally show focal slowing [4]. CSF analysis typically demonstrates elevated opening pressure, lymphocytic pleocytosis, elevated protein, absence of infectious causes, and negative oligoclonal bands and IgG index [7]. Management largely involves symptomatic treatment of headache and reassurance of disease course [8]. Most cases resolve within months and the frequency and severity of attacks traditionally diminish over time.

Here, we report a case of a patient with HaNDL presenting atypically with a thunderclap headache.

## 2. Case Report

A 32-year-old woman with a recent diagnosis of transient ischemic attack (TIA) presented to the emergency department 2.5 hours after awakening from sleep with sudden onset, thunderclap headache described as the worst headache of her life. Her headache was frontal, pressure-like, exacerbated by supine positioning, and was associated with nausea, vomiting, and photophobia. The headache reached maximal intensity within 60 seconds of onset. She denied prior history of headaches and migraines and denied recent viral illness and/or infectious symptoms. On exam, her vitals were normal and she had no focal neurologic deficits. A noncontrast CT of the head was negative for subarachnoid hemorrhage (SAH) and intracerebral hemorrhage (ICH). MRI brain revealed no evidence of stroke or blood products, but was notable for hydrocephalus and mega cisterna magna, presumed to be chronic given normal head circumference and the absence of transependymal flow. MR angiography of head and neck was negative for findings to support reversible cerebral vasoconstriction syndrome (RCVS) or dissection, and MR venogram was negative for cerebral venous sinus thrombosis (CVST). Intravenous normal saline, magnesium, and metoclopramide provided relief of headache.

She reported an admission to an outside hospital five days prior for a progressive syndrome of migratory paresthesias followed by global aphasia and right hemiparesis. During that admission, she was treated with tissue plasminogen activator given her aphasia and right hemiparesis. While in the intensive care unit, her neurological deficits resolved within 6 hours of symptom onset, but she developed a moderate intensity migrainous headache with mild photophobia and nausea. This headache resolved by the next day. Brain MRI was negative for stroke and MR angiography of head and neck was unrevealing for a dissection or other vessel pathology. Additional workup was also negative including 24 hours of video EEG monitoring, a transesophageal echocardiogram, and arterial and venous hypercoagulability studies. She was discharged on aspirin 81 mg daily for presumed transient ischemic attack.

We performed a lumbar puncture in the lateral decubitus position with extension of her lower extremities showing an opening pressure of 28 mmH_2_O, 28/uL total nucleated cells with 99% lymphocytes, 1/uL red blood cell, 33.2 mg/dL total protein, 64 mg/dL glucose, negative xanthochromia, negative bacterial and fungal culture, negative cryptococcus antigen, and negative meningoencephalitis viral panel.

The patient was discharged with a diagnosis of HaNDL. Her prior diagnosis of TIA was misdiagnosed, as her initial symptoms were consistent with HaNDL syndrome. Her aspirin was discontinued. Over the next 8 weeks after discharge, she developed periodic right-sided paresthesias followed by migrainous headaches of moderate severity with photophobia and nausea that would subside within 60–120 minutes. For the headaches, she used abortive agents such as acetaminophen, nonsteroidal anti-inflammatory drugs, and metoclopramide. These episodes became less intense and less frequent over the ensuring 8 weeks until they completely abated.

## 3. Discussion

We report a rare case of HaNDL presenting with thunderclap headache. Thunderclap-quality headache is defined as a severe and sudden onset headache, which reaches maximal intensity in one minute or less [9]. The most common causes of thunderclap headache include aneurysmal SAH and RCVS [10, 11]. Other less common causes of thunderclap headache are cervical or intracranial arterial dissection, CVST, pituitary apoplexy, posterior reversible vasoconstriction syndrome, primary intracerebral hemorrhage, and intracerebral hemorrhage secondary to ruptured cavernous malformations or arteriovenous malformations [11].

HaNDL typically manifests with gradual onset migrainous headaches and rarely presents as thunderclap headache as in our case [7]. In a single-center study of 433 patients who presented to the hospital with acute headache complaints (including thunderclap headache), only 1 patient was diagnosed with HaNDL after workup [12]. Furthermore, in a systematic review of 219 journal articles of causes of thunderclap headache other than SAH, only 1 case report described HaNDL [13].

Our case highlights the importance of recognizing HaNDL as a potential cause of thunderclap headache in the setting of negative workup for neurologic emergencies.
